# Longitudinal associations among insomnia, stigma, social support, and depressive symptoms in tuberculosis: evidence from RI-CLPM and LGCM

**DOI:** 10.1186/s12889-026-27992-z

**Published:** 2026-06-15

**Authors:** Xiangmin Liu, Huanhuan Li, Xinyu Bai, Zihan Chen, Huizhen Lan, Yujia Zhao, Mei Feng, Shu Gong, Xiangdong Tang

**Affiliations:** 1 Department of Pulmonary and Critical Care Medicine, West China Hospital, Sichuan University/West China School of Nursing, Sichuan University, Chengdu, China; 2 Mental Health Center, National Center for Mental Disorders, West China Hospital, Sichuan University/West China School of Nursing, Sichuan University, Chengdu, China; 3 Department of Intensive Care Unit, The Fourth People Hospital of Nanning, Nanning, China; 4 Department of Nursing, West China Hospital, Sichuan University/West China School of Nursing, Sichuan University, Chengdu, China; 5 Sleep Medicine Center, Mental Health Center, National Center for Mental Disorders, Sleep Research Laboratory, West China Hospital, Sichuan University, Chengdu, China

**Keywords:** Tuberculosis, Depressive symptoms, Insomnia, Stigma, Social Support, Random Intercept Cross-lagged Panel Model, Latent Growth Curve Models

## Abstract

**Background:**

Depression is highly prevalent and severe in people with tuberculosis (PWTB), often linked to insomnia, stigma, inadequate social support, and other psychological factors. However, the longitudinal and dynamic relationships between depression and these factors remain unclear, as most existing evidence stems from cross-sectional studies. We therefore examined these associations using random intercept cross-lagged panel model (RI-CLPM) and latent growth curve models (LGCMs).

**Methods:**

This study was conducted in three hospitals in China (West China Hospital, Guangyuan Mental Health Center, and the Fourth People’s Hospital of Guangxi) from October 2022 to January 2023. Using convenience sampling, we carried out a 6-month longitudinal study with assessments at treatment initiation (T1), one month (T2), two months (T3), and six months (T4). Measures included depressive symptoms, insomnia, stigma, social support, anxiety, and perceived stress. The data were analyzed using SPSS 26.0 and Mplus 8.3. RI-CLPM was used to examine the within-person bidirectional relationship between insomnia and depressive symptoms. LGCMs were used to analyze how stigma, anxiety, stress, and social support were associated with the trajectory of depressive symptoms over time.

**Results:**

The study enrolled 266 participants (166 men and 100 women; mean age 48.1 ± 18.0 years). The RI-CLPM showed significant within-person stability for both insomnia and depressive symptoms across time points (autoregressive paths: *β* = 0.236–0.458 for insomnia; *β* = 0.135–0.454 for depressive symptoms; all *P* < 0.05). The cross-lagged paths revealed mutual associations: higher insomnia was associated with subsequent depressive symptoms at T1→T2, T2→T3, and T3→T4 (*β* = 0.334, 0.572, 0.209; all *P* < 0.05), while depressive symptoms were linked to later insomnia at T1→T2 and T3→T4 (*β* = 0.378 and 0.164; *P* < 0.05) but not at T2→T3 (*β* = -0.094, *P* = 0.086). The LGCMs indicated that higher levels of stigma (across T1–T4), anxiety (at T1, T2, and T4), and perceived stress (at T1) were positively associated with concurrent depressive symptoms (all *P* < 0.05), and greater social support was associated with a faster decline in depressive symptoms over time (*P* < 0.05).

**Conclusion:**

Insomnia is associated with subsequent depressive symptoms in PWTB, suggesting sleep-focused interventions as a promising target. Stigma consistently covaries with depressive symptoms, while greater social support is linked to faster recovery, underscoring the need to address both stigma and support in TB depression management.

## Introduction

Tuberculosis (TB) remains a leading infectious killer worldwide, with an estimated 10 million new cases and more than one million deaths annually [1]. Depression is highly comorbid with TB; a recent meta-analysis reported a pooled incidence of 45.19% (95% CI: 38.04–52.55) [2]. Co-occurring depression in people with TB (PWTB) impairs immunity and medication adherence, reducing treatment success and increasing mortality and transmission risks [3, 4]. For example, Keneilwe et al. reported that delayed TB treatment was more prevalent among depressed participants (42.6% vs. 18.8%) [5], and Abdul et al. reported a 4.46-fold greater risk of treatment failure in depressed PWTB, which increased to 34.5-fold if depression persisted after the intensive phase [6].

Depression in PWTB is embedded in an interconnected network of psychological and biological factors [7]. A key node in this network is insomnia, a well-established, modifiable risk factor for depression [8, 9], and its impact may be amplified by inflammation or stress [10]. Although neurobiological mechanisms exist, our study focuses on clinical and behavioral correlates. Given that insomnia disrupts emotion regulation and circadian rhythms, its assessment and management should be a clinical priority [11]. Moreover, meta-analytic evidence indicates that heightened stigma and poor social support are core psychosocial risk factors for depression in PWTB [12], with anxiety [13] and perceived stress [14] also showing significant associations. However, most existing evidence is cross-sectional, which cannot elucidate temporal dynamics, directionality, or longitudinal course. This gap is particularly critical for understanding the insomnia–depression link in TB, where inflammatory states and fluctuating psychological distress across treatment phases [15] may complicate this relationship. Therefore, a longitudinal examination of how these variables interact over time is methodologically and clinically imperative.

To address these gaps, we conducted a 6-month longitudinal study using two advanced modeling approaches: a random intercept cross-lagged panel model (RI-CLPM) to examine within-person temporal relationships between insomnia and depressive symptoms, and latent growth curve models (LGCMs) to analyze how stigma, anxiety, stress, and social support are associated with the trajectory of depressive symptoms. Based on the literature, we propose two hypotheses: H1. At the within-person level, insomnia and depressive symptoms would be bidirectionally related over time (tested with RI-CLPM). H2. Higher stigma, anxiety, and perceived stress would be associated with more severe concurrent depressive symptoms and a worse trajectory, whereas greater social support would correspond to a more favorable course (tested with LGCM). This study provides actionable longitudinal evidence to inform targeted psychosocial interventions in TB care.

## Methods

### Participants

Eligible participants with initial drug-susceptible TB were recruited from West China Hospital, Guangyuan Mental Health Center, and the Fourth People’s Hospital of Guangxi from October 2022 to January 2023 using convenience sampling. Depressive symptoms, insomnia, stigma, social support, anxiety, and stress were assessed at treatment initiation (T1, inpatient) and after 1 month (T2, outpatient), 2 months (T3, outpatient), and 6 months (T4, outpatient). These time points were aligned with the TB treatment protocol, which lasts at least 6 months with a two-month intensive phase (isoniazid, rifampicin, pyrazinamide, ethambutol) followed by a four-month continuation phase (rifampicin, isoniazid) [16]. The study was approved by the Biomedical Research Ethics Committee of West China Hospital, Sichuan University.

The inclusion criteria for the study were: (1) recently diagnosed with TB, following the National TB Program guidelines [17]; (2) age 14 years or older; (3) receiving first-line anti-TB drugs (isoniazid, rifampicin, pyrazinamide, and ethambutol); (4) adequate communication and cognitive skills to complete the questionnaire; and (5) informed consent (or assent from minors with parental consent) and follow-up participation.

The exclusion criteria were: (1) diagnosed with psychiatric or severe physical disorders, including but not limited to tumors, critical infections, and multiorgan failure; (2) comorbid communicable diseases such as acquired immune deficiency syndrome (AIDS) or syphilis; (3) special populations such as pregnant women; (4) experiencing critical incidents affecting sleep or mental health such as severe trauma or bereavement, at enrollment or during follow-up; and (5) loss to follow-up.

### Measures

Demographic data, including age, sex, education level, marital status, monthly income, and TB type, were gathered through an electronic structured questionnaire. Assessments of depressive symptoms, insomnia, stigma, stress, anxiety, and social support were conducted using the appropriate and standardized nine-item Patient Health Questionnaire (PHQ-9), Pittsburgh Sleep Quality Index (PSQI), TB-Related Stigma Scale (TRSS), Perceived Stress Scale (PSS), seven-item Generalized Anxiety Disorder Scale (GAD-7), and Social Support Rating Scale (SSRS), respectively. Chinese versions of these scales have been validated for clinical use, with excellent Cronbach’s alpha scores of 0.812–0.938 in this study.

#### Pittsburgh Sleep Quality Index (PSQI)

The PSQI is a 19-item self-report instrument for assessing sleep quality and sleep disturbances [18], including insomnia [19, 20], over the past month. It generates a global score ranging from 0 to 21 by summing seven component scores, with higher scores indicating poorer sleep [21]. In this study, insomnia was operationally defined as a PSQI score > 5, representing scale-identified sleep difficulties rather than a clinical diagnosis. This cutoff has high sensitivity (98.7%) and specificity (84.4%) in patients with primary insomnia [22].

#### The nine-item Patient Health Questionnaire (PHQ-9) and seven-item Generalized Anxiety Disorder Scale (GAD-7)

The PHQ-9 and GAD-7 are self-report measures based on the Diagnostic and Statistical Manual of Mental Disorders, Fourth Edition (DSM-IV) criteria and are widely used for assessing probable depression and anxiety over the preceding two weeks [7]. Both use a 4-point Likert scale (0 = “not at all” to 3 = “nearly every day”), with higher scores representing greater severity [23]. The established cutoff (PHQ-9 ≥ 10) was applied to define probable depression [24].

#### TB-Related Stigma Scale (TRSS)

The TRSS is a validated 9-item scale for TB-related stigma among Chinese patients [25]. It uses a 4-point Likert scale (0 = “strongly disagree” to 3 = “strongly agree”), with higher total scores representing greater stigma [26].

#### Perceived Stress Scale (PSS)

The PSS-10 is a widely used measure of perceived stress over the past month [27]. Items are rated on a 5-point Likert scale from 1 to 5, with items 4, 5, 7, and 8 reverse-scored. Total scores range from 10 to 50, with higher scores indicating greater perceived stress.

#### Social Support Rating Scale (SSRS)

The SSRS is a 10-item scale developed for Chinese populations [28] that covers three dimensions: subjective support, objective support, and utilization of support. Total scores range from 12 to 66, with higher scores reflecting greater perceived social support.

### Sample size calculation

We performed power analyses using powRICLPM and semPower.powerLGCM (4 time points, α = 0.05), assuming small-to-moderate cross-lagged effects (β = 0.15–0.25) and moderate model complexity (two constructs, autoregressive/cross-lagged paths, within-time correlations). The results showed that a sample size of 250–300 achieved 0.8 power; our obtained sample (*N* = 266) was sufficient.

### Statistical analysis

All scale scores were analyzed as continuous variables in the main models (RI-CLPM and LGCM); cutoff values (e.g., PHQ-9 score ≥ 10) were used only for descriptive characterization or secondary dichotomization. Descriptive analyses were conducted using SPSS (version 21.0). Continuous variables are presented as means ± standard deviations, and categorical variables as frequencies and percentages. Missing follow-up data were handled by generating twenty (m = 20) imputed datasets using Markov chain Monte Carlo, including all longitudinal measures (depressive symptoms, insomnia, stigma, anxiety, and stress) in the imputation model. The imputation model also included fully observed baseline covariates (age and sex) as auxiliary variables to improve precision. Missing at random (MAR) was assumed because attrition was unrelated to observed baseline characteristics (all *P* > 0.05). For the subsequent SEM analyses in Mplus, each imputed dataset was analyzed separately using the MLR estimator (maximum likelihood with robust standard errors), and the parameter estimates were pooled across the 20 datasets using Rubin’s rules as implemented in Mplus (which pools estimates automatically).

A random intercept cross-lagged panel model (RI-CLPM) was constructed in Mplus 8.3 to examine the prospective, directional relationships between the PSQI and PHQ-9 scores. Unlike traditional cross-lagged models, the RI-CLPM accounts for stable between-person differences through latent random intercepts, thereby isolating within-person dynamics. In this framework [29], observed scores are decomposed into stable between-person traits and time-specific within-person states. The within-person component models two types of effects: autoregressive paths, which estimate an individual’s subsequent deviation in one variable from their prior deviation, and cross-lagged paths, which test whether a deviation in one variable (e.g., insomnia) predicts a subsequent deviation in another (e.g., depression), after controlling for prior levels. This allows for an examination of temporal dynamics at the within-person level, independent of stable between-person differences. In the RI-CLPM, all autoregressive and cross-lagged paths were freely estimated and thus allowed to vary over time. We chose not to impose equality constraints across waves, as this would make untested assumptions about temporal stationarity; freely estimating each path is the standard and more flexible approach in the RI-CLPM.

Furthermore, conditional latent growth curve models (LGCMs) were employed to examine the longitudinal associations of perceived stigma, anxiety, stress, and social support with the trajectory of depressive symptoms. In the LGCM, stigma, anxiety, and stress were treated as time-varying covariates, and social support as a baseline predictor (measured only at T1). This structural equation framework parameterizes change through an intercept (initial level), a linear slope (constant rate of change per unit time), and a quadratic slope (acceleration or deceleration rate), with the latter indicating the curvature of the developmental trajectory [30]. In the LGCM, time was coded as the number of months since baseline: T1 = 0 (baseline), T2 = 1 (1-month follow-up), T3 = 2 (2-month follow-up), and T4 = 6 (6-month follow-up). This coding reflects the actual measurement intervals, allowing the linear slope to be interpreted as the average monthly change in depression scores and the quadratic slope to represent the acceleration or deceleration of this change. Model fit was assessed using the comparative fit index (CFI), root-mean-square error of approximation (RMSEA), and standardized root mean squared residual (SRMR). A model was considered acceptable if CFI > 0.90 and RMSEA and SRMR < 0.08 [29, 31]. A p-value of < 0.05 was considered statistically significant. To determine the optimal functional form for the depressive symptom trajectory, we fitted three conditional growth models: linear, quadratic, and freed-curve. The freed-curve model did not converge properly (estimated slope variance was negative) and was therefore excluded from further comparisons. The remaining two models (linear and quadratic) were then compared, with lower Akaike information criterion (AIC) and sample-size-adjusted Bayesian information criterion (BIC) values indicating superior fit.

## Results

### Demographic characteristics and prevalence

This longitudinal study initially enrolled 300 participants with newly diagnosed tuberculosis (all had complete baseline data). The follow-up completion rates were 96.7% (*n* = 290) at T2, 93.0% (*n* = 279) at T3, and 88.7% (*n* = 266) at T4. Among those retained, item-level missingness was < 5% for all scales at all time points. During follow-up, 34 participants were excluded due to loss of contact or refusal to continue participation (see exclusion criteria). The remaining 266 participants (166 men, 100 women) aged 14–86 years (mean 48.1 ± 18.0) comprised the final analytic sample (see Table 1); more than 90% had pulmonary TB. To assess attrition bias, baseline characteristics were compared between completers and those lost to follow-up; no significant differences were found (all *P* > 0.05), suggesting random attrition.

**Table 1:** Baseline characteristics of the participants (*N* = 266)

Item	Category	*N* (%) or Mean ± SD
Age, years		48.1 ± 18.0
Male	Male	166 (62.4%)
	Female	100(37.6%)
Education	Junior high school or below	175 (65.8%)
	High school / secondary vocational school	53 (19.9%)
	Junior college or above	38 (14.3%)
Marital Status	Unmarried	28 (10.5%)
	Married	218 (82.0%)
	Divorced or widowed	20 (7.5%)
Monthly income	≤ 1000	131 (49.2%)
	1000–3000	70(26.3%)
	>3000	65(24.4%)

*Mean ± SD* Mean ± standard deviation

From T1 to T4, the average PHQ-9 and PSQI scores tended to decrease. The number of participants with probable depression (PHQ-9 score ≥ 10) was 62, 44, 32, and 27, corresponding to a decrease in prevalence from 23.3% to 10.1%. Similarly, the number of participants with insomnia (PSQI > 5) was 154, 126, 113, and 98, with the prevalence decreasing from 57.9% to 36.8%. Pearson correlation analysis revealed significant relationships between variables (PSQI and PHQ-9 scores) at different times (*P* < 0.05). This met the requirements for cross-lagged analyses (see Table 2).

**Table 2:** Descriptive statistics and correlations for the PSQI and PHQ-9 scores by time point

Item	Mean ± SD	PSQI T1	PSQI T2	PSQI T3	PSQI T4	PHQ9 T1	PHQ9 T2	PHQ9 T3	PHQ9 T4
PSQI T1	9.3 ± 3.7	1.000							
PSQI T2	6.0 ± 4.0	0.745*							
PSQI T3	4.7 ± 2.8	0.409*	0.561*						
PSQI T4	2.5 ± 2.0	0.308*	0.430*	0.453*					
PHQ9 T1	10.1 ± 5.7	0.729*	0.791*	0.440*	0.345*				
PHQ9 T2	6.5 ± 4.1	0.767*	0.759*	0.380*	0.361*	0.855*			
PHQ9 T3	4.1 ± 2.8	0.580*	0.753*	0.432*	0.466*	0.606*	0.643*		
PHQ9 T4	2.7 ± 1.7	0.394*	0.496*	0.529*	0.345*	0.430*	0.421*	0.562*	1.000

T1 = Time 1(baseline), T2 = Time 2 (1 month), T3 = Time 3 (2 months), and T4 = Time 4 (6 months)

*PSQI* Pittsburgh Sleep Quality Index, *PHQ-9* Patient Health Questionnaire-9, *Mean ± SD* Mean ± standard deviation

* *P* < 0.05

### Bidirectional relationship between depressive symptoms and insomnia from the RI-CLPM

**Fig. 1:**
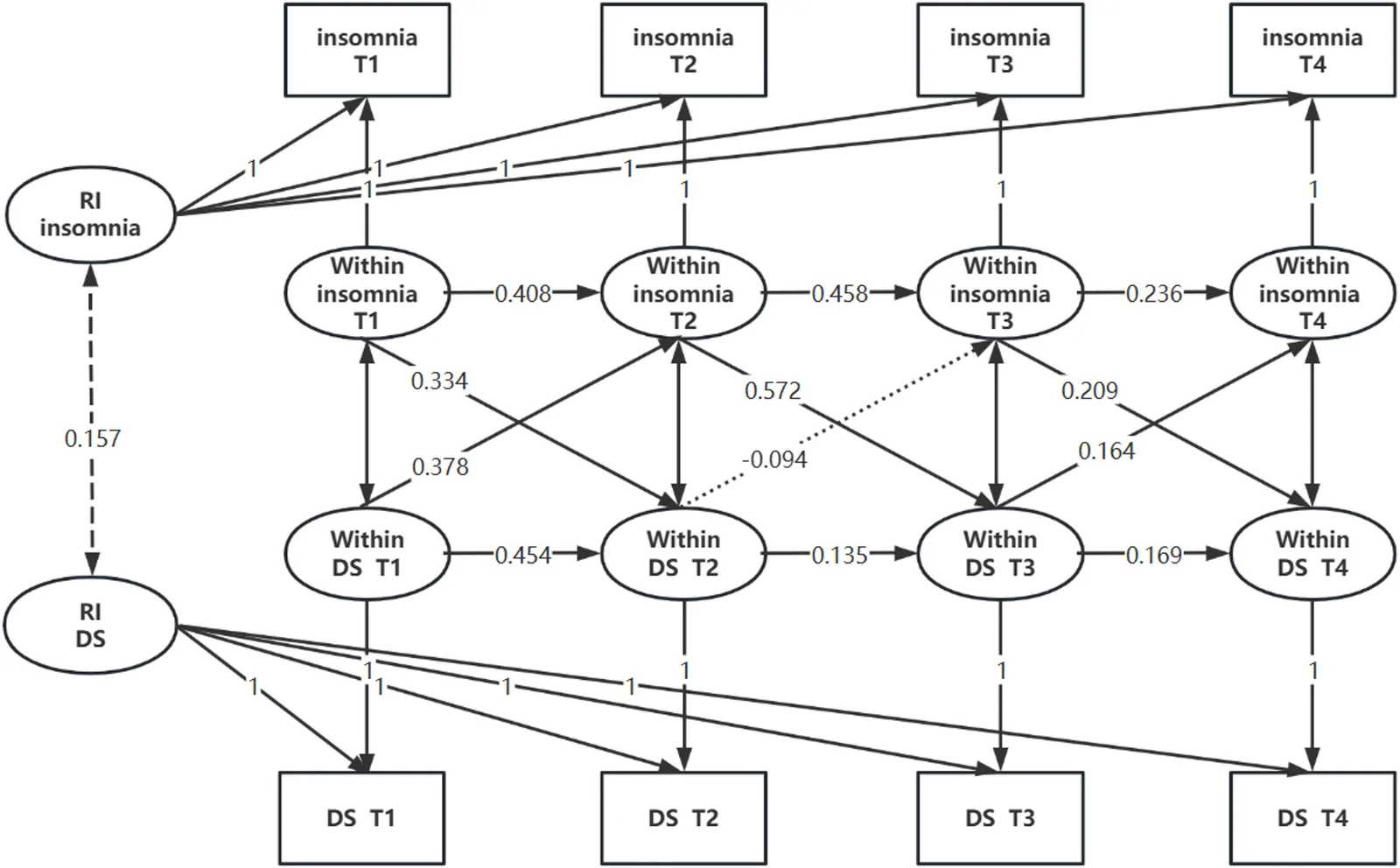
RI-CLPM of depressive symptoms and insomnia among PWTB in different periods. Notes: T1 = Time 1 (baseline), T2 = Time 2 (1 month), T3 = Time 3 (2 months), and T4 = Time 4 (6 months); DS: depressive symptoms. Numbers indicate standardized regression coefficients (*β*). The solid arrows indicate significant effects (*P* < 0.05), and the dotted arrows indicate non-significant effects (*P* > 0.05)

After controlling for between-person differences, the RI-CLPM results revealed a statistically significant bidirectional relationship between within-person insomnia and within-person depressive symptoms, as most cross-lagged paths were significant (*P* < 0.05). The model demonstrated excellent fit to the data: χ² = 6.522, df = 9, CFI = 1.000, RMSEA = 0.000, SRMR = 0.010, with all fit indices meeting the established thresholds for good fit. The autoregressive paths revealed significant within-person stability for both insomnia and depressive symptoms over time. Prior levels of within-person insomnia were significantly associated with subsequent insomnia (T1→T2: *β* = 0.408, 95% CI [0.302, 0.514]; T2→T3: *β* = 0.458, 95% CI [0.327, 0.589]; T3→T4: *β* = 0.236, 95% CI [0.113, 0.359]; all *P <* 0.05). Similarly, previous within-person depressive symptoms were followed by later depressive symptoms (T1→T2: *β* = 0.454, 95% CI [0.407, 0.501]; T2→T3: *β* = 0.135, 95% CI [0.027, 0.243]; T3→T4: *β* = 0.169, 95% CI [0.081, 0.257]; all *P <* 0.05). The cross-lagged paths revealed a mutual temporal relationship. Specifically, within-person insomnia was significantly associated with subsequent depressive symptoms at multiple intervals (T1→T2: *β* = 0.334, 95% CI [0.238, 0.430]; T2→T3: *β* = 0.572, 95% CI [0.435, 0.709]; T3→T4: *β* = 0.209, 95% CI [0.123, 0.295]; all *P* < 0.05). Correspondingly, within-person depressive symptoms were linked to later insomnia at T1→T2 (*β* = 0.378, 95% CI [0.298, 0.458], *P* < 0.05) and T3→T4 (*β* = 0.164, 95% CI [0.068, 0.260], *P* < 0.05), but not at T2→T3 (*β* = -0.094, 95% CI [-0.202, 0.014], *P* = 0.086) (see Fig. 1).

Notably, the cross-lagged coefficients from insomnia to depressive symptoms were numerically larger than those from depressive symptoms to insomnia, and the R² values for depressive symptoms were consistently higher than those for insomnia (see Table 3). These patterns suggest a potentially more consistent predictive role of insomnia. However, these differences were not formally tested (e.g., via Wald tests), and conclusions should be drawn cautiously. Over time, predictive efficacy decreased for both symptoms: for insomnia, R² decreased from 0.685 (T2) to 0.291 (T4); for depressive symptoms, it decreased from 0.772 (T2) to 0.408 (T4).

**Table 3:** Coefficient of determination (R²) for all regression models in the CLPM

Regression model	*R*² Estimate	SE of *R*²	*P* Value
WT2I ~ WT1I + WT1D	0.685	0.030	< 0.001
WT3I ~ WT2I + WT2D	0.327	0.046	< 0.001
WT4I ~ WT3I + WT3D	0.291	0.080	< 0.001
WT2D ~ WT1I + WT1D	0.772	0.031	< 0.001
WT3D ~ WT2I + WT2D	0.574	0.055	< 0.001
WT4D ~ WT3I + WT3D	0.408	0.066	< 0.001

WT1I, WT2I, WT3I, and WT4I represent the potential insomnia severity at baseline, 1 month, 2 months, and 6 months of PWTB, respectively, as estimated after controlling for individual patient effects using a random intercept model. WT1D, WT2D, WT3D, and WT4D represent the potential depression at baseline, 1 month, 2 months, and 6 months of PWTB, respectively, after controlling for individual levels

*SE* Standard error

### Associations of perceived stigma and social support with depressive symptoms from LGCMs

**Fig. 2:**
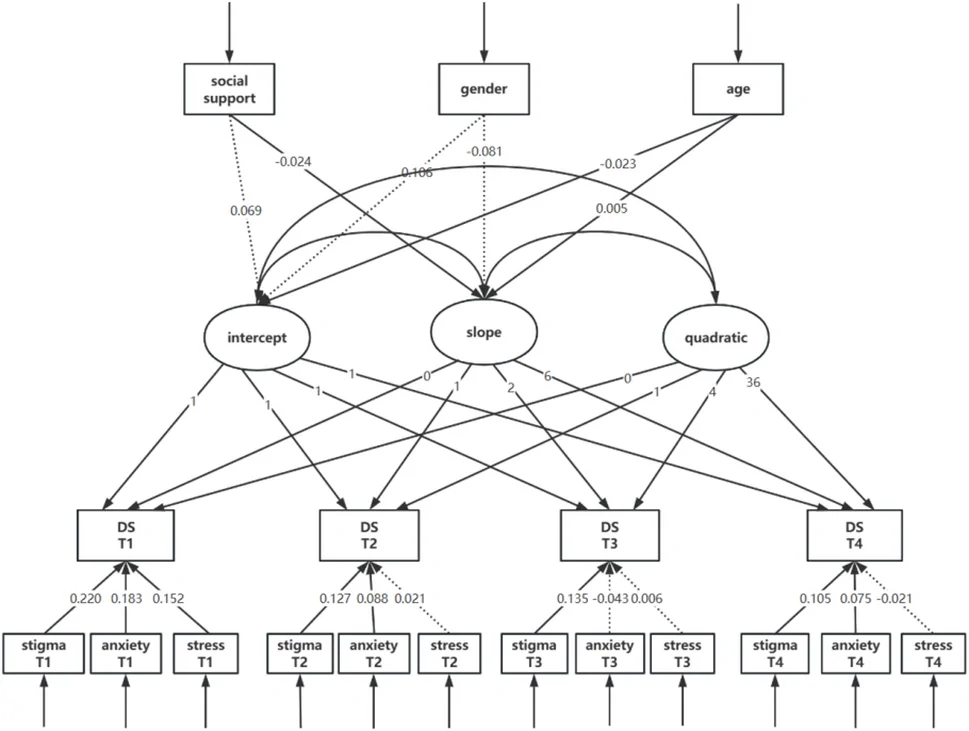
Associations of covariates (social support, stigma, anxiety, and stress) with depressive symptoms in PWTB. Notes: DS: depressive symptoms; T1 = Time 1 (baseline), T2 = Time 2 (1 month), T3 = Time 3 (2 months), and T4 = Time 4 (6 months). Numbers indicate standardized regression coefficients (*β*). The solid arrows indicate significant effects (*P* < 0.05), and the dotted arrows indicate non-significant effects (*P* > 0.05)

Both linear and quadratic conditional growth models were fitted to the depressive symptoms trajectory. The linear model showed poor fit (CFI = 0.689, RMSEA = 0.159, SRMR = 0.137), whereas the quadratic model demonstrated acceptable fit (CFI = 0.907, RMSEA = 0.058, SRMR = 0.036). Accordingly, the quadratic model was retained for further analysis. Its parameter estimates describe a trajectory with the highest severity of depressive symptoms at baseline (intercept = 3.888, *P* < 0.05; covariate-centered, raw mean = 10.1), a significant initial decrease (linear slope = -1.380, *P* < 0.05), and a decelerating rate of change over time (quadratic slope = 0.251, *P* < 0.05). This pattern indicates that depressive symptoms improve most rapidly during the early treatment phase, with the pace of improvement gradually slowing. Significant individual variation was observed in both the initial level of depressive symptoms and the rate of change (all variance components *P* < 0.05). Consistent with the Methods, stigma, anxiety, and stress were modeled as time-varying covariates, and social support as a baseline predictor (measured only at T1). After controlling for confounders such as age and sex, social support was not significantly associated with the initial level of depressive symptoms (*β* = 0.069, *P* = 0.334) but showed a significant negative relationship with its rate of change (*β* = -0.024, *P* < 0.05). This suggests that greater social support corresponded to faster symptom reduction over time. Perceived stigma showed consistent positive associations with concurrent depressive symptoms from T1 to T4 (*β* = 0.105–0.220, all *P* < 0.05). Anxiety was positively associated with depressive symptoms at T1, T2, and T4 (*β* = 0.075–0.183, all *P* < 0.05), whereas perceived stress was correlated with depressive symptoms only at T1 (*β* = 0.152, *P* < 0.05). Detailed results are presented in Table 4 and Fig. 2.

**Table 4:** Model fit and coefficients for the three LGCMs of depressive symptoms in PWTB

Item	Linear LGCM	Quadratic LGCM
χ2 (df)	364.307 (47)	344.309 (45)
AIC	5026.197	5010.198
BIC	5122.951	5114.120
P	< 0.001	< 0.001
CFI	0.689	0.907
RMSEA (90% CI)	0.159 (0.144, 0.175)	0.058(0.043, 0.074)
SRMR	0.137	0.036
Coefficients		
Intercept	3.173*	3.888*
Slope	0.306	-1.380*
Quadratic		0.251*
Variances		
Intercept	12.651*	13.596*
Slope	0.356*	0.170*
Quadratic		0.016*

*AIC* Akaike information criterion, *BIC* Bayesian information criterion; the smaller the AIC and BIC values are, the better the model fit, *CFI* Comparative fit index, *RMSEA* Root mean square error of approximation, *SRMR* Standardized root mean squared residual, *90% CI* 90% confidence interval

*P < 0.05

## Discussion

Our longitudinal analysis revealed the dynamic interplay of psychological factors during the first six months of TB treatment. First, a bidirectional relationship was observed between insomnia and depressive symptoms, with a more consistent pathway from insomnia to subsequent depressive symptoms. Second, perceived stigma was consistently correlated with depressive symptoms, whereas greater social support was linked to faster recovery. Third, depressive symptoms were most severe at baseline and tended to decrease non-linearly over time.

### Insomnia and subsequent depressive symptoms in PWTB

Depression and insomnia often cooccur and share a bidirectional relationship [32, 33]. Robust evidence has revealed insomnia as an independent risk factor for depression onset, worsening, and recurrence [9, 34]. Mechanistically, persistent sleep disruption may contribute to mood disorders through multiple pathways, including overactivation of the stress system, dysregulation of inflammatory and monoaminergic systems, and impairments in circadian rhythms and emotion regulation mediated by prefrontal cortex activity [11]. As stress-related disorders, insomnia and depression are characterized by maladaptive coping mechanisms [35]. Individuals with insomnia perceive negative events as more stressful and employ less effective coping strategies [36], which in turn can interfere with falling asleep. Moreover, sleep deprivation may intensify the perception and internalization of stigma [37], promoting social avoidance [38]. Similarly, depression increases vulnerability to insomnia through heightened stress sensitivity, thus completing a bidirectional cycle [39].

Our RI-CLPM analysis extends this evidence to the TB context, suggesting that within-person insomnia is associated with subsequent depressive symptoms across the entire six-month follow-up (T1→T2→T3→T4). The cross-lagged coefficients from insomnia to depressive symptoms were consistently significant across all three intervals, whereas those from depressive symptoms to insomnia were significant only at T1→T2 and T3→T4, with a non-significant path at T2→T3 (β = -0.094, *P* = 0.086). These findings suggest a more consistent role of insomnia in relation to subsequent depressive symptoms, further supported by the higher R² values for depressive symptoms (see Table 3). This pattern is clinically plausible in the context of TB: treatment-specific stressors, including fear of infection, cough, chest pain, and neurostimulatory adverse effects of medications such as isoniazid [40], may disrupt sleep and thereby exacerbate the insomnia-to-depression pathway. However, these differences were not formally tested; thus, conclusions should be drawn cautiously. The single non-significant cross‑lagged path (depressive symptoms T2 → insomnia T3) could hypothetically reflect temporary decoupling during the intensive phase of TB treatment (months 1–2). One possible hypothesis is that the dual effects of isoniazid [40]—its monoamine oxidase-inhibiting property—may rapidly alleviate depressive symptoms, while its central nervous system excitatory effects impair sleep maintenance—might lead to a transient disruption of the depression-to-insomnia link. Alternatively, this null finding could be due to the relatively small sample size. We emphasize that these are speculative and require further testing. Importantly, this isolated non-significance does not undermine the overall bidirectional pattern observed across the remaining time intervals.

This strong association between insomnia and subsequent depressive symptoms highlights the need to integrate sleep-focused strategies into early TB care. This imperative is further supported by meta-analytic evidence of a dose-response relationship in which improvements in sleep quality led to better mental health outcomes [41]. Therefore, we recommend the systematic integration of sleep assessment into initial TB care to enable early identification of insomnia. This should be followed by timely interventions, such as sleep hygiene education, brief cognitive behavioral therapy for insomnia (CBT-i), or pharmacotherapy, to disrupt the pathogenic cycle. However, implementing these interventions in routine TB care is often challenging because of limited mental health resources, high caseloads, and financial barriers, especially in resource-limited settings. Task-shared strategies (e.g., brief sleep advice in nursing care) or e-CBTI (digital cognitive-behavioral therapy for insomnia) may offer more feasible alternatives. Proactively addressing sleep health from the beginning of treatment may reduce the incidence and severity of comorbid depression in this vulnerable population.

### Stigma covaries with depressive symptoms over time in PWTB

In addition to biological mechanisms such as the inflammatory cascade, psychosocial factors, especially stigma, are significant contributors to depression in PWTB [4]. Similar to HIV-related stigma, TB-related stigma is rooted in societal labeling, negative stereotypes, and discrimination [42], primarily driven by fear of infection, historical associations with poverty, and misconceptions [43]. It is estimated that 42%-82% of PWTB experience such stigma to varying degrees [44], manifested as avoidance by others, barriers to healthcare access and employment, and overt social marginalization or rejection. Such experiences of exclusion and devaluation are quantitatively linked to depression: A meta-analysis revealed that individuals who perceived TB-related stigma had a significantly greater risk of depression (OR = 4.13, 95% CI: 1.41–12.09) [12]. Fear of being stigmatized leads many individuals to avoid or delay TB diagnosis, which in turn contributes to later treatment initiation, poorer adherence, unfavorable treatment outcomes, and increased community transmission [44–46]. Our LGCM results further indicate a positive association between stigma and depressive symptoms from T1 to T4, demonstrating their consistent covariation throughout the follow-up period. This sustained association underscores the clinical necessity of addressing stigma throughout the entire treatment course. Previous analyses of intervention pathways for reducing TB stigma have proposed a framework that simultaneously targets key groups: PWTB, the general population, and healthcare providers [46, 47]. For PWTB and their families, interventions should focus on psychosocial support and peer leadership to counter internalized and anticipated stigma. For the general public, education and norm-shifting aim to reduce enacted or community-level stigma. For healthcare providers, efforts should address institutional stigma through improved infection-control communication and organizational culture change. Among these, information-based interventions at the community and household levels are crucial, as they reduce both enacted and internalized stigma by improving public knowledge and dispelling misconceptions about TB [43]. This evidence underscores that addressing stigma is not only a clinical imperative but also a feasible public health goal, achievable through coordinated strategies that engage patients, communities, and health systems alike.

Anti-TB treatment, including long treatment cycles and various drug side effects, along with economic burden and social bias, can cause significant stress. This stress can lead to anxiety and insomnia. More than half of PWTB report high perceived stress [14], which leads to adverse coping behaviors such as social isolation, a factor known to worsen perceived stigma [48]. The most frequently reported social stressors among PWTB include stigma, discrimination, isolation, and lack of social support [49]. Furthermore, the absence of robust social support significantly increases their vulnerability to depression [12], which may be due to feelings of neglect, isolation, and worthlessness [14]. Notably, our LGCM analysis further revealed that social support was negatively linked to the progression rate of depressive symptoms, indicating its role in facilitating recovery. This protective function may be attributed to the capacity of strong social support to mitigate negative feelings by providing critical care, economic support and emotional resources, thereby helping patients cope with illness-related distress and reducing psychological distress [17, 50]. The established role of social support as a mediator between stigma and depression in other populations further validates it as a crucial intervention target [51]. Importantly, the present longitudinal findings validate and extend our prior cross-sectional symptom network analysis, which identified insomnia as a bridge symptom and negative family perceptions as crucial mediators [7]. This longitudinal evidence thereby strengthens the empirical foundation for establishing these factors as high-efficacy intervention targets. Addressing them could not only resolve depressive symptoms but also synergistically dismantle the broader insomnia–depression–anxiety–stigma complex.

### Depressive symptom trajectory and the early treatment phase as a critical window for intervention

Our LGCM analysis revealed a decelerating decline in depressive symptoms over the 6-month follow-up (quadratic slope = 0.251, *P* < 0.05), with the most rapid improvement during the intensive treatment phase (first 2 months, T1→T3) and slower gains thereafter (T3→T4), likely due to early symptom relief from treatment. This pattern aligns with previous reports that psychological distress is elevated during early treatment [52, 53]. Specifically, perceived stress and anxiety at baseline (T1) were positively associated with concurrent depressive symptoms, reflecting the profound distress commonly experienced at TB diagnosis [53]. Excessive or prolonged stress activates the hypothalamic‒pituitary‒adrenal (HPA) axis to release glucocorticoids, which can disrupt sleep [54] and promote depression-like behavior [55]. Moreover, the RI-CLPM showed that depressive symptoms at one time point were significantly followed by depressive symptoms at the next (T1→T2, T2→T3, T3→T4), indicating strong stability over time. Therefore, the early treatment period represents a critical window for both screening and intervention. Effectively preventing severe depressive symptoms requires timely, customized strategies initiated during this phase, informed by patient-specific characteristics and early assessments.

Together, these findings suggest that depressive symptoms in PWTB are shaped by multiple factors. Insomnia had the strongest and most consistent relationship with later depressive symptoms. Stigma was steadily associated with depressive symptoms over time, whereas social support was linked to faster recovery. Anxiety and stress were most noticeable around the time of diagnosis and decreased thereafter. This suggests that interventions targeting sleep, stigma, and social support together may work better than focusing on any one of them alone.

## Limitations

Our study has several limitations. First, our convenience sampling approach and the exclusion of participants who experienced serious incidents during follow-up (aimed at confounding control) may have biased our sample toward a more stable cohort, limiting the generalizability of our findings to patients facing significant concurrent stressors. Additionally, clinical characteristics related to TB severity (e.g., lung involvement, bacterial burden) were not systematically captured, which limits interpretation of whether the observed psychological trajectories partly reflect disease burden rather than psychosocial processes alone. Second, our findings are primarily based on self-reported symptom measures rather than definitive clinical diagnoses. Constructs such as stigma and social support were examined as a whole without breaking down their finer components, which may have missed the unique contributions of each dimension. Formal measurement invariance across time points was not tested; thus, cross-time comparisons should be interpreted with caution. Third, the significant autoregressive pathways in the RI-CLPM suggest stable variance in the constructs of insomnia and depressive symptoms in addition to fluctuating trait variance, potentially complicating the estimation of pure cross-lagged effects between their states. Fourth, given the exploratory nature of this study, we did not adjust for multiple comparisons. We focus on effect sizes and confidence intervals, interpret p-values cautiously, and note that traditional corrections (e.g., Bonferroni) are overly conservative for non-independent parameters in SEM. Finally, the small sample size and brief follow-up period limited the comprehensive longitudinal analysis; a larger sample with longer follow-up would yield more precise and robust results.

## Conclusion

This study found that insomnia was associated with subsequent increases in depressive symptoms in people with tuberculosis, suggesting that sleep-focused interventions may represent a promising intervention target. Stigma also showed a persistent association with depressive symptoms throughout treatment, while greater social support was linked to faster recovery. These findings support prioritizing insomnia assessment and management as a primary focus for addressing TB-related depressive symptoms, with stigma reduction and social support enhancement as complementary approaches.

## Data Availability

The data used to support the findings of this study are not publicly available due to their proprietary and confidential nature as records of West China Hospital, Sichuan University. However, they can be made available upon reasonable request from the corresponding author.

## Abbreviations

PWTB: People with tuberculosis
TB: Tuberculosis
PHQ-9: Patient Health Questionnaire-9
PSQI: Pittsburgh Sleep Quality Index
RI-CLPM: Random Intercept Cross-lagged Panel Model
LGCMs: Latent growth curve models
DS: Depressive symptoms
